# Liver sampling: a vital window into HBV pathogenesis on the path to functional cure

**DOI:** 10.1136/gutjnl-2017-314873

**Published:** 2018-01-13

**Authors:** Upkar S Gill, Laura J Pallett, Patrick T F Kennedy, Mala K Maini

**Affiliations:** 1 Department of Hepatology, Centre for Immunobiology, Blizard Institute, Barts and the London School of Medicine and Dentistry, Queen Mary University of London, London, UK; 2 Division of Infection and Immunity, UCL, London, UK

**Keywords:** cccDNA, fine needle aspirate, hepatitis B, hepatic fibrosis, histopathology, liver immunology, tissue resident cells, liver biopsy

## Abstract

In order to optimally refine the multiple emerging drug targets for hepatitis B virus (HBV), it is vital to evaluate virological and immunological changes at the site of infection. Traditionally liver biopsy has been the mainstay of HBV disease assessment, but with the emergence of non-invasive markers of liver fibrosis, there has been a move away from tissue sampling. Here we argue that liver biopsy remains an important tool, not only for the clinical assessment of HBV but also for research progress and evaluation of novel agents. The importance of liver sampling has been underscored by recent findings of specialised subsets of tissue-resident immune subsets capable of efficient pathogen surveillance, compartmentalised in the liver and not sampled in the blood. Importantly, the assessment of virological parameters, such as cccDNA quantitation, also requires access to liver tissue. We discuss strategies to maximise information obtained from the site of infection and disease pathology. Fine needle aspirates of the liver may allow longitudinal sampling of the local virus/host landscape. The careful utilisation of liver tissue and aspirates in conjunction with blood will provide critical information in the assessment of new therapeutics for the functional cure of HBV.

## The backdrop

Viral hepatitis remains the seventh most common cause of death worldwide, with mortality increasing sharply in the last decade to levels on the scale of HIV, tuberculosis or malaria.^1^ The dramatic recent progress in hepatitis C treatment means that this infection can now be cured with short courses of direct-acting antivirals, shifting the impetus to better case-finding and enhancing accessibility to these effective but expensive drugs. By contrast, hepatitis B can still only be cured in a tiny minority of the estimated 240 million people chronically infected with this virus. Reducing new infections by vaccination and other preventative measures remains of paramount importance and in this regard, it is welcome news that the UK joined other EU nations with universal hepatitis B virus (HBV) vaccination of all neonates in 2017. For those already infected with HBV, there is the prospect of better treatments on the horizon, with efforts and expectations in academia and industry being dramatically intensified in response to progress in the fields of hepatitis C virus (HCV) infection and cancer. There is now a worldwide call for wider access to existing suppressive therapies, to be followed by attempts to achieve the state of functional cure seen following natural resolution of acute infection, with minimal residual virus kept under long-term immune control without the need for ongoing antiviral drugs.^2 3^

**Figure 1:**
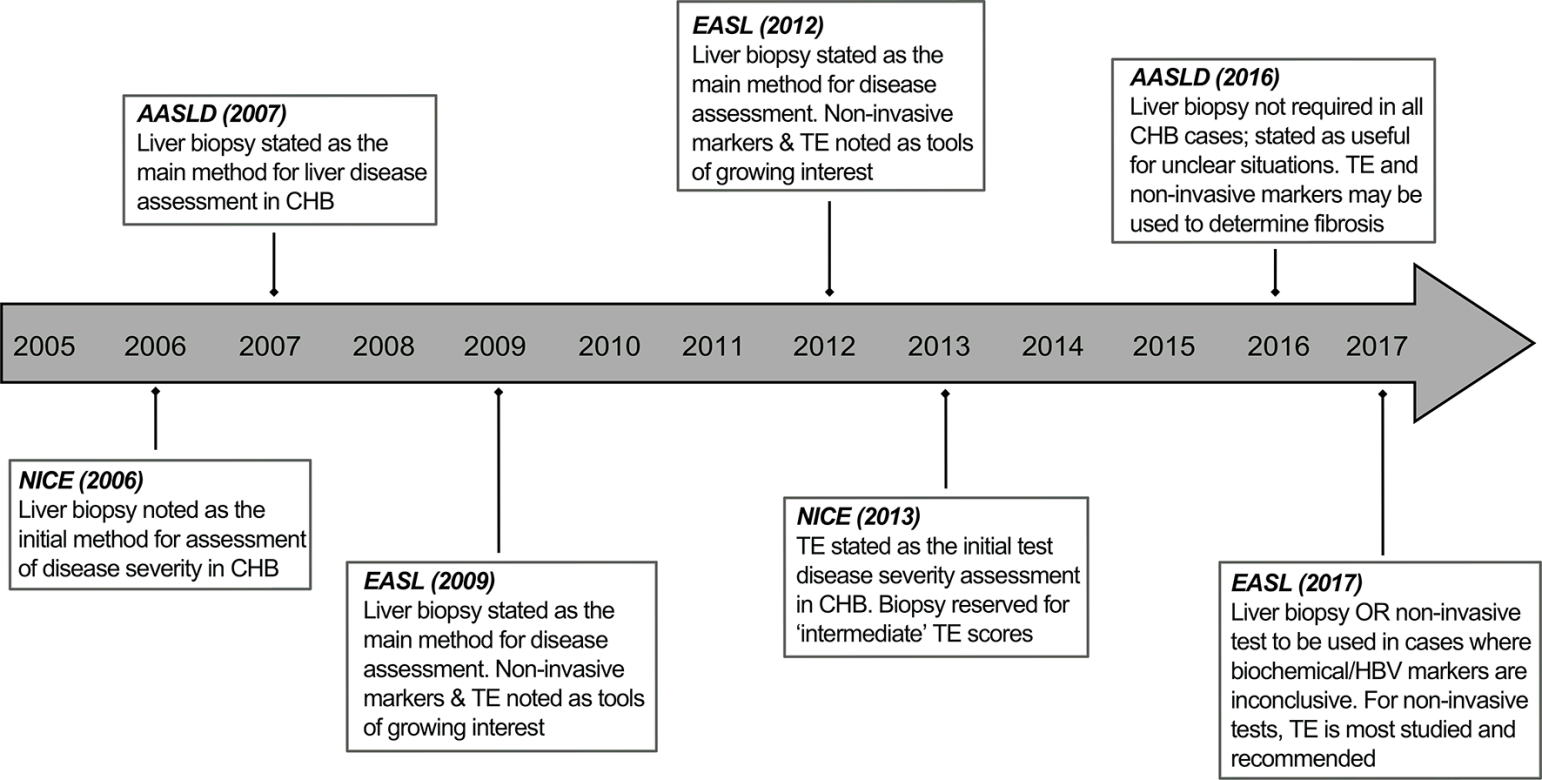
Historical progression of the modality of liver disease assessment as outlined in national and international clinical practice guidelines for hepatitis B virus. CHB, chronic hepatitis B; NICE, National Institute for Health and Care Excellence; TE, transient elastography.

HBV is an hepatotropic virus, so the site of viral replication and disease pathogenesis is within the liver. Traditionally, a mainstay of management has therefore included assessment of liver histology using tissue obtained by a needle core biopsy. This remains the most accurate way of definitively assessing the degree of liver fibrosis for initial HBV disease staging and of ruling out other causes of liver disease. There is a strong case for the continued assessment of liver histology in combination with serological markers for treatment decisions, particularly in ambiguous cases. In addition, their use may instigate other clinical investigations (eg, gastroscopy to exclude portal hypertension), in cases where advanced liver disease is detected. Liver biopsy is, however, no longer the standard of care in a number of clinics due to the advent of non-invasive methods of fibrosis assessment.^4 5^ The use of transient elastography (TE) and serum markers to assess fibrosis, along with indirect assessment of liver inflammation/damage by serum transaminases and quantitation of circulating hepatitis B viral load and antigens is increasingly being regarded as an adequate alternative. This is reflected in recent updates to national and international clinical practice guidelines in which TE is recommended as the first-line modality for disease assessment, with liver biopsy reserved for indeterminate cases (figure 1).^6–13^ The move away from liver biopsies in routine HBV assessment is likely influenced by the fact that their use in HCV disease management has been rendered almost redundant. However, the place of liver biopsy in the management of chronic hepatitis B infection (CHB) is currently a subject of much debate.

TE, using FibroScan, allows rapid assessment of liver stiffness by employing a shear-wave to measure vibration-velocity, a technique previously used in the food industry to gauge the maturity of cheese.^14^ TE, and serum-based markers such as the enhanced liver fibrosis test (ELF)^15 16^ have some advantages (table 1) including better patient acceptability and suitability for longitudinal assessment. However, their major disadvantage is limited validation and lower diagnostic accuracy in HBV, particularly with coincident inflammation or steatosis.^5 17 18^ Liver biopsy remains the ‘gold standard’ for the definitive assessment of HBV disease stage (table 1) and the availability of different techniques (Box) allows selection of the most appropriate for each case in order to minimise the small overall risk. Liver biopsy, as discussed below, is subject to sampling error and should, ideally, be complemented by non-invasive assessments conducted in parallel and then used for follow-up for the clinical management of HBV.

**Table 1:** Advantages and disadvantges of liver biopsy sampling, transient elastography and non-invasive markers of fibrosis

	Liver biopsy	TE and non-invasive markers of fibrosis
Advantages	Definitive histological diagnosis* Allows exclusion of other liver diseases* Accurate fibrosis stage* Additional assessment of necroinflammatory reaction and steatosis* Validated score for HBV* Helpful for delineation in intermediate disease* Surplus tissue and slides stored for retrospective analysis*† Tissue availability for routine HBV virological assessment (HBsAg staining)*† Tissue availability for state-of-the-art virology (eg, cccDNA, integrated DNA)**†** Tissue availability for state-of-the-art immunology research (eg, resident lymphocytes, HBV-specific T cells)**†**	Minimal risk* Easy to perform* Lower cost per test* Painless, good patient acceptability Immediate results available at ‘point of care’‡* Easily repeated, allows longitudinal assessment*
Disadvantages	Invasive, bleeding risk (0.01% mortality)* Pain-related morbidity, variable patient acceptability* Sampling error* Contraindicated in certain cases* High cost per test*	Costly equipment‡* Technical expertise required‡* Unreliable in obese patients‡* Skewed results with deranged LFTs‡* Optimal cut-off levels not validated in HBV*

*Relevant for clinical purposes.

**†**Relevant for research purposes.

‡Relevant to TE only.

cccDNA, covalently closed circular DNA; HBV, hepatitis B virus; HBsAg, hepatitis B surface antigen; LFT, liver function test; TE, transient elastography.

In this article, we argue that liver biopsy remains an important tool in the majority of patients with HBV infection, for clinical disease assessment and for research progress and evaluation of novel agents (table 1). Even in cases where the information gleaned may not directly alter patient management, it is pivotal for the optimisation of future therapeutics, and an argument can therefore be made to seek ethical approval for such sampling. We review new findings on compartmentalisation within the liver, including a population of tissue-resident T cells capable of acting as local specialists in immune defence. On the basis of the many crucial aspects that cannot be assessed in the blood, we propose that liver biopsy should remain part of disease assessment in CHB and be prioritised in the evaluation of new strategies for functional cure of HBV.

Box **Types of liver biopsy**

- Percutaneous biopsy:
  - Palpation/percussion guided or image-guided; transthoracic approach most common, subcostal approach may be used*
- Transvenous biopsy:
  - Transjugular/transfemoral approach. Required in patients with clotting abnormalities and ascites and for indications where free/wedge hepatic vein pressure measurements are required.
- Laparoscopic/surgical biopsy:
  - May be performed at time of surgical intervention. Liver can be directly visualised thus tissue yield is good, but general anaesthesia is required.
- Coaxial biopsy:
  - Needle puncture device through which smaller needle biopsies are performed. Multiple passes through this approach can be performed.

*Percutaneous biopsies can be performed with core aspiration needles; Menghini, Jamshidi or Klatskin. Usually performed with 16G needle which provides better cores, 18G needles may be used which may be better accepted by patients, but cores may be more fragmented. Sheath cutting needles may also be used (Tru-cut), usually smaller gauge, but sample may be less fragmented.

## What we can sample in the blood

### Immune parameters

**Figure 2:**
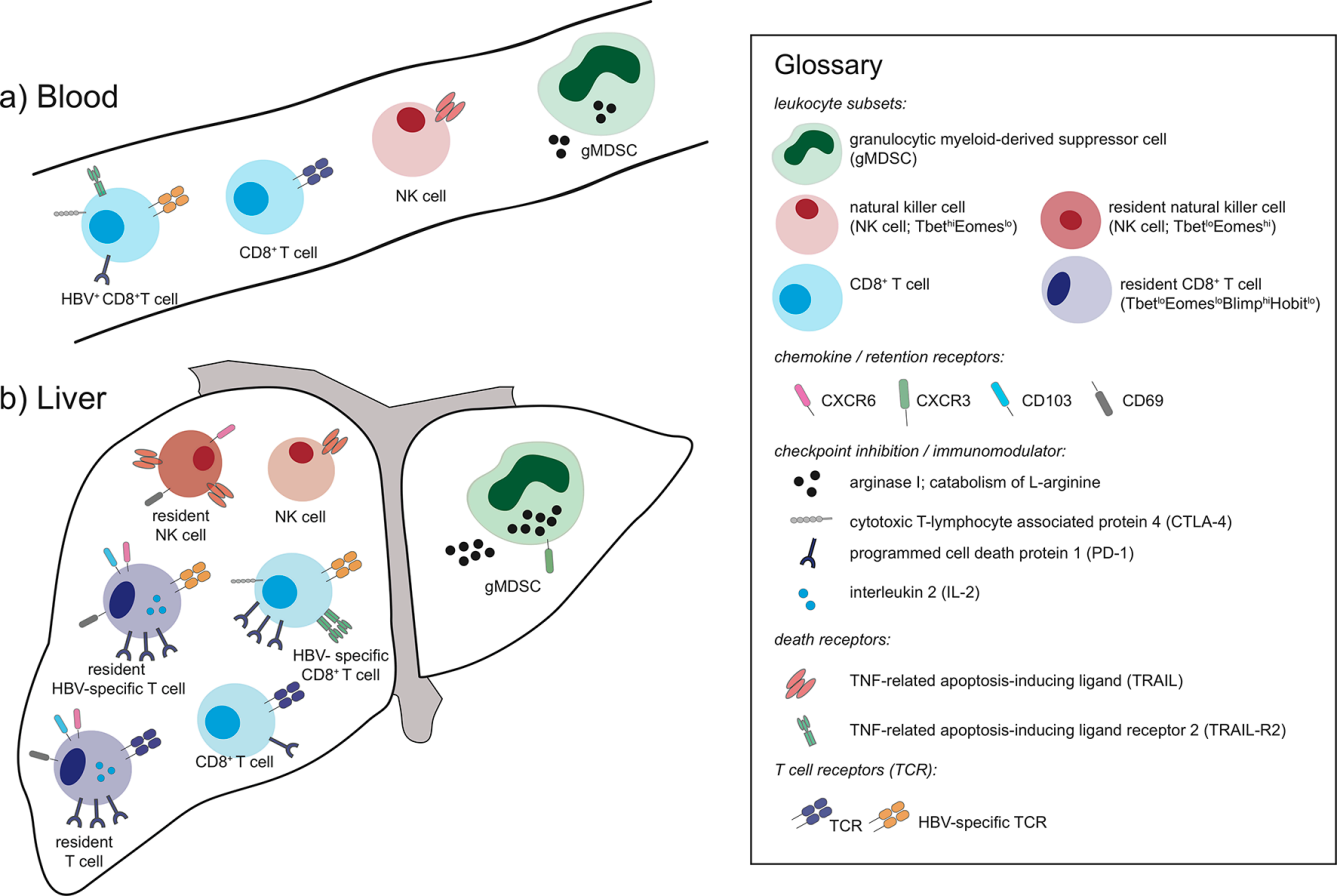
Schematic of A) peripheral and B) intrahepatic lymphocyte populations depicting examples that can be sampled from both sites but have an exagerated phenotype in the liver and others that are unique to the liver.

**Figure 3:**
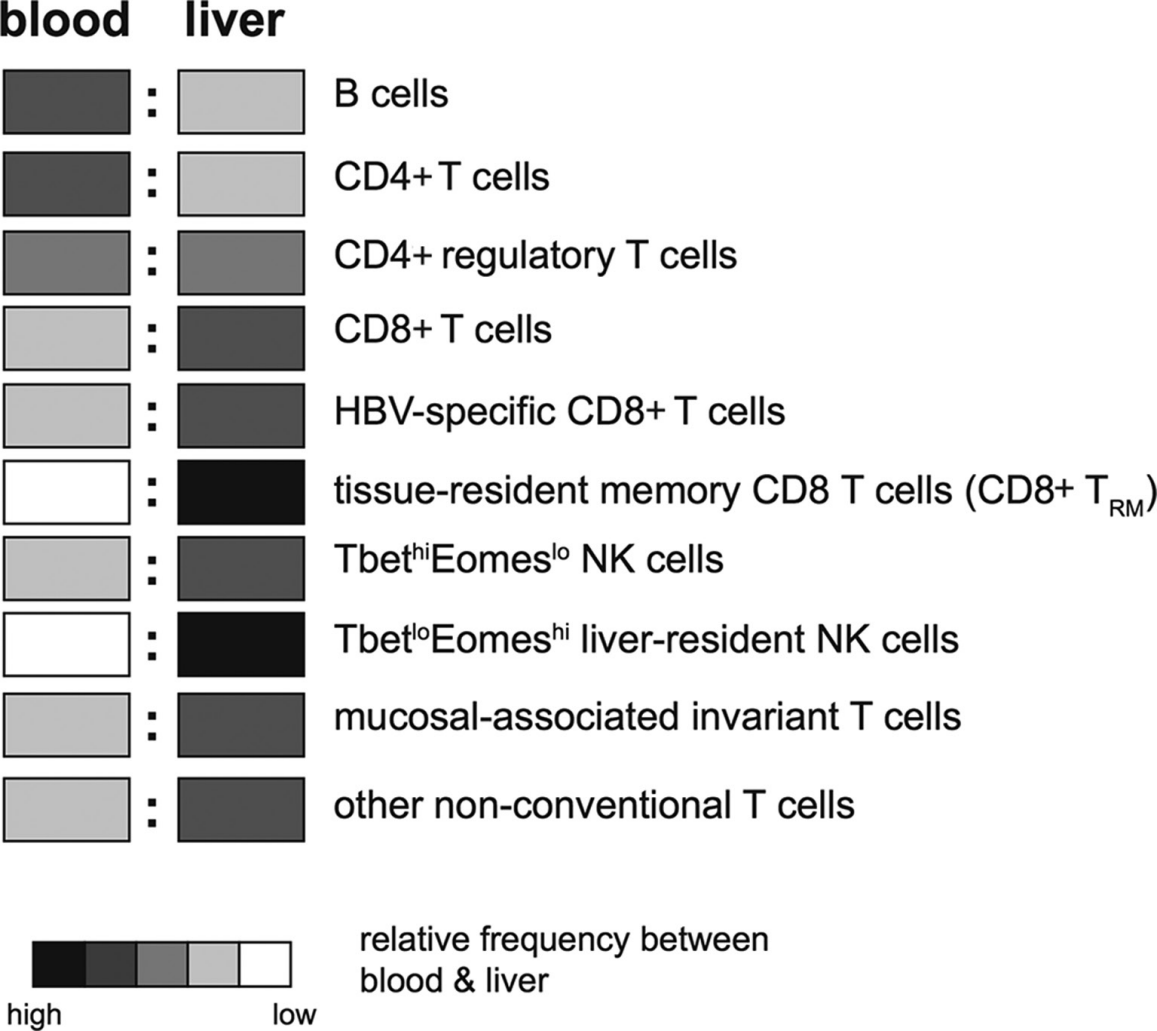
Relative frequencies of lymphocyte subsets between the blood and the liver (key indicates relative abundance between the two sites, not between different cell types).

Most studies of HBV immune responses in humans have relied solely on blood sampling; many clear-cut changes have been detectable and useful insights into disease pathogenesis have been made. In a few studies, responses studied thoroughly in the periphery have been compared with those within intrahepatic lymphocytes by paired sampling of the liver in a small subset of donors.^19–27^ Until recently, such comparisons had generally validated blood monitoring, showing that findings made from the periphery were representative of their intrahepatic counterparts, with the same responses and features simply being present in a more exaggerated form in the liver (figures 2 and 3). HBV-specific CD8 T cells, critical for antiviral control, were found to be present at higher frequency in the liver than the blood but retained the same inverse correlation between frequency and viral load in both sites.^19 24^ It has been possible to define important mechanisms constraining these antiviral responses within the blood, such as their expression of the coinhibitory molecule programmed cell death protein 1 (PD-1), the death receptor, TNF-related apoptosis-inducing ligand receptor 2 (TRAIL-R2) and the oxidative stress ligand MHC class I polypeptide-related sequence A  (MICA), although these changes became far more evident when examining their intrahepatic counterparts.^24 26 28 29^

Similarly, we were able to detect an expansion of granulocytic myeloid-derived suppressor cells correlating with disease activity in the circulation of patients with CHB, although in the liver this population was further expanded, expressing a more immunoregulatory phenotype (increased arginase I and degranulation, marked by increased surface expression of CD63).^30^ Other immune responses, such as B cells, are not enriched in the liver and, along with antibody responses, are easily studied in the periphery^31^; however, their further evaluation in the liver remains important in light of work suggesting they can play local pathogenic roles in HBV.^32^ Crucially, as discussed below, it has now become evident that some specialised immune populations are completely compartmentalised in the liver, making the blood only one component of an adequate assessment of HBV immunity.

### Virological parameters: emerging surrogates of covalently closed circular DNA in the periphery

The study of covalently closed circular DNA (cccDNA), the episomal viral genome of HBV within hepatocyte nuclei, remains of critical importance as extensive efforts are made to develop therapeutic approaches to degrade or silence it. A major barrier to progress in the field has been the inability to accurately measure cccDNA in blood and assays for its determination in the periphery are likely to face many hurdles.^33^ While serum HBV DNA and hepatitis B envelope antigen (HBeAg) status remain the foundation of virological assessment in today’s clinic, they provide limited information on cccDNA.^34–36^ This has led to a rigorous search for surrogates of cccDNA in the periphery.^37^ Progress in this sphere will be dependent on access to liver tissue to validate the utility of any putative new marker. In recent years, quantitative HBsAg (qHBsAg), which is readily measured in the serum, has been proposed as a robust measure of cccDNA transcription. However, recent findings suggest that in patients with HBeAg-negative disease and those who have received long-term nucleos(t)ide analogues (NAs), integrated DNA, rather than cccDNA, is a major source of circulating HBsAg.^38 39^ In addition, the quantity of HBsAg measured in the serum as free HBsAg has the possibility of being confounded by the presence of immune complexes with coexisting anti-HBs, or the emergence of pre-S/S variants preventing HBsAg release from hepatocytes, thus giving an unreliable measurement of total HBsAg.^40 41^ Future assays will aim to distinguish the small, medium and large constituent proteins of HBsAg, as well as that derived from cccDNA versus integrated DNA.

More recently, hepatitis B core-related antigen (HBcrAg) has emerged as a potentially more accurate surrogate for cccDNA than qHBsAg. Assays for ‘core-related antigen’ measure denatured HBeAg, HBcAg and the pre-core protein (aa28 to aa15)^42^ using a chemiluminescent enzyme immunoassay and reflect the sum of complete virions (with rcDNA or viral RNA), empty virions and secreted HBeAg. When HBV DNA becomes undetectable on NA therapy, HBcrAg should better reflect the quantity and transcriptional activity of cccDNA than qHBsAg, since it is unlikely to be produced by integrated DNA. In line with this, studies have shown that HBcrAg correlates with intrahepatic cccDNA both in treatment naïve patients^43^ and in those treated with NAs,^44^ although these findings have yet to be validated across all patient groups and genotypes.

HBV RNA can also be measured in the serum. It is packaged within nucleocapsids and both full-length and truncated RNA forms can circulate in patients.^45^ Prior to HBeAg seroconversion, HBV RNA levels are thought to fall and thus can predict HBeAg seroconversion.^46^ Levels of HBV RNA are also presumed to reflect intrahepatic cccDNA transcription. During NA therapy, pregenomic RNA from ongoing cccDNA transcription can no longer be reverse transcribed and instead accumulates and is encapsidated; secreted levels may serve as a predictor of viral rebound.^47^ However, no commercial assays are available for detecting HBV RNA levels and thus its utility as a surrogate for intrahepatic cccDNA remains unclear at this juncture.

## What we are missing in the blood

### Histology

Histology provides a more detailed assessment of the liver, reflecting cumulative damage over time in addition to ongoing inflammation. A recent meta-analysis of 683 patients has shown that even with slight increases in alanine aminotransferase (ALT), a substantial number of patients (up to 50%) have significant fibrosis.^48^ Moreover, in patients with normal serum ALT and levels of HBV DNA between 2000 and 20 000 IU/mL (so-called grey-zone patients), significant levels of fibrosis have been reported.^49^ We have previously shown that patients considered ‘immune tolerant’ (HBeAg+chronic infection) had levels of fibrosis similar to those found in patients labelled immune active (HBeAg±chronic hepatitis), notwithstanding differences in biochemical and virological parameters.^50^

Validated histological scoring systems are used in HBV; Ishak, Knodell and METAVIR being routinely used to assess disease activity.^51^ However, the potential for sampling error and interobserver/intraobserver variability may lead to inaccuracy of fibrosis staging from liver biopsy alone. New methods of tissue image analysis to detect collagen proportionate area^52^ and collagen scoring by novel tools such as second harmonic imaging^53^ will reduce subjectivity. A liver biopsy can additionally be used to identify the coexistence of other diseases; in particular coincident steatosis and non-alcoholic steatohepatitis, an increasingly common comorbidity, would be indistinguishable from HBV-related liver inflammation without a biopsy.^54^ The ability to score interface hepatitis, confluent necrosis, portal inflammation, focal lytic necrosis, apoptosis and focal inflammation, which constitute the histological activity index/necroinflammatory index, is unique to histological assessment of liver tissue.^55^

Beyond diagnostic evaluation, additional immunohistochemical staining can be undertaken, providing novel insights into HBV immunopathogenesis. Combining histochemical analysis of liver injury with specialised stains of paraffin-embedded sections for immune cell distribution can provide valuable insights into HBV pathogenesis. For example, interleukin (IL)-17-producing CD4 T cells were seen to accumulate in the lobular and portal areas of livers with HBV-related injury, suggesting a role in disease pathogenesis.^56^ Localisation of natural killer (NK) cells in areas of hepatic necrosis implicated them in HBV-related liver damage^57^; NK cells expressing the death ligand TRAIL, and hepatocytes expressing the death-inducing receptor TRAIL-R2, were both increased on histological staining of HBV-infected livers, pointing to a pathogenic role for this pathway.^21^ Much remains to be learnt from more in-depth histology, to assess the topological relationship between viral particles, immune infiltrates and disease pathology using newly available multiparameter analysis, as discussed below.

### Immunological features of the liver

The liver has an immunological composition which is distinct from blood or other lymphoid and non-lymphoid organs. Its inherent cellular composition is strikingly different, and even when circulating cell types pass through the liver they may be differentially shaped by the local milieu of cytokines, nutrients and hypoxia; these influences are likely to be exaggerated by the sluggish blood flow through the narrow-lumen vascular bed of the sinusoids.^58^ In addition to the parenchymal cells (hepatocytes) that HBV replicates within, the liver contains unique non-parenchymal cell types including specialised scavengers/antigen-presenting cells such as liver sinusoidal endothelial cells and Kupffer cells (liver-specific macrophages) that can influence neighbouring lymphocytes.^58 59^ Although Kupffer cells are the largest macrophage population in humans, they have been difficult to study due to lack of adequate markers to distinguish them from liver-infiltrating monocytes.^60^ They contribute to the tolerogenic environment provided by the liver, by producing immunosuppressive cytokines such as IL-10 and transforming growth factor-β (TGF-β).^61^

The intrahepatic lymphocyte population is very different from blood (figure 3); for example, the liver is selectively enriched for CD8 rather than CD4 T cells, a reversal of the situation in the blood. While classical Tregs (CD25+FoxP3+CD4+) have been identified in both blood and liver of patients with CHB,^62–65^ an atypical regulatory population of CD25-FoxP3+CD4+T cells has been found to be unique to the liver.^66^ The large population of CD8 T cells in the liver is dominated by memory subsets, with significantly less naïve CD8 T cells than blood.^67^ NK cells are much more prevalent in the liver than other peripheral tissues (with the exception of the uterus),^68^ accounting for up to 40% of all intrahepatic lymphocytes.^57 69^ More recently, it has been recognised that mucosal-associated invariant T cells are another innate-like cell that preferentially accumulate within the liver, at higher frequencies than either the blood or gut.^70^ In addition to their T cell receptor (TCR)-dependent recognition of MHC class I-related molecule-presented bacterial riboflavin derivatives, they can be activated in a TCR-independent manner by viral infection or toll-like receptor (TLR)-8 agonists to produce substantial quantities of interferon-γ (IFN-γ)^71 72^; as with NK cells, this requires accessory cell production of IL-12/IL-18.^73^ Other non-conventional T cells enriched within the liver include γδ-T cells.^74^

### Immunological compartmentalisation in the liver

We have characterised a novel population of tissue-resident memory T cells (T_RM_) that can persist long term in the human HBV-infected liver and do not recirculate. These CD8 T cells have specialised adaptations for maintaining antiviral surveillance within the tolerogenic liver environment. We find liver-resident CD8 T cells,^75–78^ defined by the coexpression of retention markers CD69 and CD103 and by a unique transcription factor profile (T-bet^lo^Eomes^lo^Blimp-1^hi^Hobit^lo^; figure 2), within both healthy and HBV-infected livers but never in the blood.^67^ Importantly, they are selectively increased in patients with well-controlled HBV infection, contain T cells specific for epitopes from all major HBV proteins and survive in the liver even after resolution of infection. All intrahepatic T cells express more PD-1 than those in the circulation but levels of this coinhibitory receptor are significantly higher on CD8 T_RM_. Despite this, CD8 T_RM_ are able to produce antiviral cytokines rapidly on restimulation, perhaps as a result of their high cell-autonomous IL-2,^67^ which has been shown to overcome PD-1-driven exhaustion.^79^ By contrast, liver CD8 T_RM_ have lower granzyme expression than other non-resident intrahepatic CD8 T cells, suggesting they may be adapted to favour non-cytolytic over cytolytic antiviral function in order to preserve organ integrity.^67 80^ Thus, a large fraction of HBV-specific T cells are compartmentalised within the liver, restrained by high PD-1 to avoid excessive proliferation and senescence and allow long-term persistence, yet poised for rapid frontline protection.^81^

The presence of a population of memory CD8 T cells persisting within an organ long after infection resolution, yet retaining immediate antigen-specific effector function, was originally described for a neurotropic influenza virus 20 years ago.^82^ In recent years, the concept of tissue-resident memory T cells has received a lot more attention, with elegant murine and human studies revealing their very high frequency among memory pools in many organs and their key role in both pathogen protection and disease pathogenesis.^83–85^ Many of the adaptations that we noted in liver T_RM_ such as rapid effector function despite inhibitory receptors, and low turnover, are core features shared between human T_RM_ in different tissues,^86^ whereas others, such as the capacity to patrol and remain within the sinusoids (through expression of lymphocyte functional-associated antigen-1 and CXCR6), are liver-specialised characteristics.^75–77 87 88^

The finding that a population of HBV-specific CD8 T cells with T_RM_ characteristics can survive within the liver following HBV resolution provides a blueprint for future therapeutic attempts to achieve functional cure. It remains to be seen whether the cytokine signals (sequential IL-15 and TGF-β) that we found could impose the liver-resident signature on peripheral T cells in vitro, will have therapeutic applicability.^67^ Critically, it will not be possible to evaluate whether future novel immunotherapeutic strategies for HBV are harnessing or recapitulating this resident population, as recently described for a malaria vaccine,^89^ unless the liver is sampled. Likewise, it will be difficult to fully understand the constraints that persisting HBV-specific T cells have to operate under, and their range of adaptations, unless those that live exclusively in the liver are studied.

The phenomenon of specialised lymphocyte populations being retained within the liver and not amenable to sampling in the blood is not restricted to CD8 T_RM_. Tissue-resident memory CD4 T cells are also well-described in other settings and may well play a role in CHB and there are likely other subsets of liver-resident cells yet to be defined. Another liver-resident population that has come to light recently is a subset of NK cells expressing high levels of CXCR6,^90^ again with a distinctive transcriptional profile: Tbet^lo^Eomes^hi^ (figure 2).^91 92^ Analysis of human liver allografts has suggested that these NK cells can be derived from the circulating NK cell subset, then becoming imprinted by the liver to remain resident for many years.^93^ This population is present in healthy and diseased livers at highly variable frequencies; the factors determining their frequency and physiological roles remain to be determined. In the setting of HBV infection they are able to express the death ligand TRAIL (figure 2), an inducible defence system allowing them to kill hepatocytes, stellate cells and HBV-specific T cells.^21 26 69 94^ In support of the latter, in situ analysis by immunohistochemistry of HBV sections from liver biopsies allowed the visualisation of NK cells in close proximity to T cells within the liver sinusoids.^26^ Antiviral and immunotherapeutic strategies used in HBV, particularly those inducing IFN-α, can potently expand and activate NK cells,^95 96^ with NAs having limited impact on these cells.^96 97^ It will again be informative to sample the liver in order to see whether these and novel therapies differentially modulate liver-resident NK cells and their antiviral and immunoregulatory potential. These targeted analyses of selected immune cells have begun to be complemented by comprehensive approaches, comparing peripheral and intrahepatic transcriptome/proteome profiling to detect additional features of HBV in an unbiased manner.^98–100^

### Virological compartmentalisation

As discussed above, sampling of liver tissue is required for the direct measurement of cccDNA and its transcriptional activity as existing surrogates measured in the circulation have substantial limitations. There are limited data on the impact of therapy on the decay of cccDNA^101 102^ and accurate future assessment of novel therapies on cccDNA will still ideally require liver biopsies.

Furthermore, the detection of integrated DNA and its response to novel therapies, an additional therapeutic goal under consideration, also relies on liver sampling.^50^ Integration of HBV DNA occurs through a process of illegitimate recombination by host enzymes acting on double stranded linear DNA,^103 104^ not required for productive replication. Integrated sequences cannot provide an adequate template for productive replication, but can produce HBsAg. Previously, the majority of research regarding integrated HBV DNA focused on its potential to drive hepatocellular carcinoma (HCC), however, recent work has shown that HBV DNA integration appears to occur early during infection and is detectable in patients considered to be in the so-called immune-tolerant disease phase (HBeAg+chronic infection).^50^ The presence of integrated HBV DNA, its role in clonal hepatocyte expansion and events leading to hepatocarcinogenesis require further investigation, underscoring the need for more studies based on human tissue samples.^105^ Furthermore, the evaluation of current drugs and novel therapies, their impact on HBV DNA integration and the modulation of cancer risk in CHB will require the study of liver tissue. Until new biomarkers are devised that are capable of identifying patients at risk of HCC development, liver biopsy remains an indispensable tool in the pursuit for HBV cure.

Along with the evaluation of cccDNA and integrated HBV DNA in the infected-liver, the presence, quantification and localisation of HBV antigens at the site of infection remains vital to further augment our understanding of immunopathogensis, and translation through to HBV therapies. Our previous work has shown the differentiation of disease phase based on HBcAg localisation, with redistribution from the nucleus to the cytoplasm as liver damage occurs. The mosaic distribution of HBV antigens might reflect different virological/immunological features, which merit further characterisation.^50^ These data are supported by a recent study underlining the link between HBcAg expression, HBV replication and higher cccDNA content within hepatocytes.^106^ Recent work has indicated that HBV antigens may not be adequately stained using conventional immunohistochemical methods^107^ and further work using liver tissue is required to optimise their detection, using techniques such as in situ hybridisation probes.^106^

## A way forward

How can we reconcile the need to sample the liver with the growing tendency to circumvent liver biopsy in the routine management of CHB? It is clear that much remains to be learnt about the histological, virological and immunological aspects of this disease that are restricted to the liver itself. This need is particularly pertinent when assessing new therapies or combinations; in order to learn as much as possible about why these may fail and how they can be further refined, it is vital to maximise the information gleaned by including samples from the site of infection and pathology. The new findings of liver-resident populations of T and NK cells serve to underscore this paradigm. Much more remains to be learnt about adaptations of lymphocytes to the liver environment and their potential for therapeutic manipulation. Similarly, the discovery that HBV antigens and nucleic acids have distinct spatial distribution highlights the value of more sophisticated in situ analysis of virological events at the single cell level using liver sections of patients treated with novel antiviral agents.^106^ Liver biopsies will continue to provide that vital telescope onto the battlefield. On the other hand, it is natural that patients and clinicians favour non-invasive assessments wherever possible.

Perhaps a good compromise for the immediate future will be to ensure that liver biopsies at least continue to be carried out in cases where the risk/benefit ratio is favourable for patients because histological assessment will help to guide clinical management. Examples of this include assessing the need to commence antiviral therapy in HBeAg-negative hepatitis with borderline or discrepant liver enzymes and elastography results or in patients who may have subtle liver disease despite fulfilling classical serum criteria of ‘immune tolerance’.^108^ Many clinicians consider that a biopsy is still justifiable to assess disease stage in the majority of patients with CHB. Since it has been shown that multiple passes do not increase the risk of complications,^109^ it makes sense to ensure that sufficient tissue is obtained to allow thorough histological assessment, and state-of-the-art assays for compartmentalised viral and immunological parameters such as cccDNA and HBV-specific T cells. In some cases, flow cytometric analysis could be supplemented by unbiased techniques such as RNA-sequencing (RNA-Seq) and proteomics/metabolomics to learn more about the liver milieu as well as its cellular constituents.

A setting where there is a particularly strong argument to sample the liver is in early phase clinical trials of novel therapies for CHB, in order to optimally define their mechanism of action and limitations. It is crucial to assess the impact of antiviral and immunomodulatory approaches on end points such as cccDNA and integrated DNA within the liver as well as proxy measures like HBV DNA and HBsAg in the periphery. If, for example, a therapeutic vaccine or checkpoint inhibitor does not achieve functional cure, it will be informative to see whether it failed to expand liver-resident HBV-specific T cells. Such assessments ideally require paired pretreatment and end-of-treatment biopsies, which may continue to be achievable in substudies with limited patient numbers. However, it must be acknowledged that the mainstay of clinical and immune monitoring of CHB trials will continue to be peripheral blood. With evolving advances in high dimensional analysis of peripheral blood mononuclear cells  (PBMC), it should ultimately be possible to identify fingerprints within the peripheral immune response that can be used as indirect biomarkers of treatment efficacy within an organ. This approach was exemplified by a recent study predicting tumour responses to PD-1 blockade using blood-based immune profiling combined with tumour imaging.^110^ A period of consolidated effort to sample blood and liver in parallel is therefore essential to delineate peripheral markers that adequately reflect intrahepatic events in CHB. Historical data from studies in chimpanzees and woodchucks where both blood and liver were sampled should also be mined to learn more about the relationship between peripheral and intrahepatic changes.^111 112^

Another solution to facilitate longitudinal assessment of novel therapies is the use of fine-needle aspirates (FNA) of the liver in place of biopsies. This technique was first shown to allow monitoring of immune parameters of CHB in 2005,^113^ but has not yet been widely adopted. It can also allow some limited assessment of hepatocytes and intrahepatic HBV antigens.^114^ Because it is rapid, painless and very safe, it can be repeated within as short an interval as 6 hours, and allows serial sampling through the course of therapy, as described recently in patients with HCV.^115 116^ At least 1 00 000 cells can be obtained, which allows several immune subsets to be examined using multiparameter flow and high dimensional analysis or even single cell PCR on sorted populations.^117 118^ We are currently investigating whether the full complement of liver-resident lymphocytes seen in tissue biopsies can also be extracted by FNA.

While FNA may prove very useful for selective immune-monitoring of CHB treatment trials, it does not allow histological staining to examine responses in situ and better elucidate HBV pathogenesis. For this, better use could be made of stored paraffin-embedded sections from the era when liver biopsies were more common in CHB. New multiplexed immunostaining, allowing automated quantitative assessment of multiple proteins on a single slide, will open up the opportunity for much more comprehensive visualisation of the topography of HBV viral particles and antigens in combination with immune infiltrates. This is vital in order to image the spatial relationship between infected hepatocytes and immune cells to better illuminate host-pathogen interactions. At present, even the proportion and distribution of infected hepatocytes and their relationship to immune infiltrates has not been defined for different phases of infection. In addition to staining for HBsAg and HBcAg, it is now possible to use highly sensitive probes for in site hybridisation of HBV RNA.^119^ Other mechanisms to enhance our knowledge of the liver-specific environment in CHB include the capacity to now culture and infect primary human hepatocytes and to use three-dimensional models recapitulating some of their interactions with, for example, Kupffer cells or stellate cells along with the addition of immune cells.^120–122^

In summary, a wave of new agents to treat HBV will be reaching clinical trials in the next few years. In order to capitalise on the opportunity to learn from these initial attempts at functional cure, and to select optimal combinations, it is imperative that we intensify efforts to sample the liver as well as blood. This need has been reinforced by recent studies highlighting unique immune responses compartmentalised within the liver, along with the hallmarks of persistent HBV: integrated and cccDNA. Available liver tissue or aspirates must be used in carefully planned virological and immunological studies, aided by new techniques that allow simultaneous assessment of many parameters. Studies including liver samples will provide vital clues to modify and combine the array of new antiviral and immunomodulatory therapies on the horizon for HBV.
